# Safety and efficacy of polydimethylsiloxane (Macroplastique®) in women with stress urinary incontinence: analysis of data from patients who completed three years follow-up

**DOI:** 10.1007/s00192-021-04827-6

**Published:** 2021-06-08

**Authors:** Gamal Ghoniem, Bilal Farhan, Mashrin Lira Chowdhury, Yanjun Chen

**Affiliations:** 1 Department of Urology, University of California Irvine (UCI), Irvine, CA USA; 2 Division of Female Urology, Pelvic Reconstructive Surgery & Voiding Dysfunction, Department of Urology, UC Irvine Health, 333 City Blvd. West, Suite 2100, Orange, CA 92868 USA; 3 Urology Division, University of Texas, Medical Branch, Galveston, TX USA; 4 Institute for Clinical and Translational Science, University of California Irvine (UCI), Irvine, CA USA

**Keywords:** Polydimethylsiloxane, Urethral bulking agent, Three years, Intrinsic sphincter deficiency

## Abstract

**Introduction and hypothesis:**

This study aimed to report 3-year completed follow-up of the safety and efficacy of Macroplastique® (MPQ) in women with stress urinary incontinence (SUI) due to intrinsic sphincter deficiency (ISD).

**Methods:**

This is a retrospective analysis of all women who completed 3-year follow-up post-MPQ injection(s) at ten medical centers. We used the ROSE registry data report of Macroplastique® [Macroplastique® Real-time Observation of Safety and Effectiveness (ROSE) registry P040050/PAS001 on 2017]. Subjective incontinence outcome and adverse effects were assessed.

**Results:**

The study included all patients (*n* = 70) who completed 3-year follow-up after the last MPQ injection. Twenty-four of 70 (34%) patients had two injections; 21/70 (30%) patients reported Stamey grade 0 and 28/70 (40%) reported Stamey grade 1. The overall patient satisfaction was 68% who completed 3-year follow-up. The composite success rate (I-QoL, PGI-S, and Stamey grade improvement) was 51.4%. No serious adverse events (AE) were reported within the completed 3-year follow-up.

**Conclusions:**

MPQ was found to be safe and efficacious for the treatment of SUI secondary to ISD in women. The overall high satisfaction rate was sustained from baseline to 3 years post-injection. Most complications were minor and transient without sequelae.

## Introduction

Stress urinary incontinence (SUI) is a major contributor to the overall disease burden of urinary incontinence with an estimated prevalence of 21–46% [1, 2]. It is characterized by involuntary leakage of urine when the intravesical pressure exceeds the urethral resistance as a result of increased intra-abdominal pressure in the absence of detrusor contraction [3]. The etiology of SUI in females has been traditionally classified into two categories: loss of anatomical support with urethral hypermobility and intrinsic urethral sphincter deficiency (ISD). This concept is now recognized to be overly simplified, and in reality, SUI is typically caused by a combination of the two in different proportions. In other words, ISD is present to varying degrees in all patients with SUI.

Urethral bulking agent (UBA) injections were the second most common procedure for the management of SUI in the Medicare population accounting for 22% of these procedures [4]. Despite the wide range of surgical options available to treat SUI, certain patients, especially those with ISD and a fixed urethra, respond poorly to surgery [5]. In 2017, a study reported a high success rate of minimally invasive procedures such as UBA for persistent or de novo SUI following suburethral sling removal [6].

The present study aimed to report the efficacy and safety of MPQ in women who completed 3 years of follow-up, using the ROSE registry data report of Macroplastique® [Macroplastique® Real-time Observation of Safety and Effectiveness (ROSE) registry P040050/PAS001 on 2017].

## Materials and methods

This is an ad hoc analysis of longitudinal data from a cohort of 70 patients enrolled in a US multicenter study (*n* = 275 by the year 2020) who completed 3 years of follow-up and were recruited between October 2008 and August 2015 using data from the end of 2017. Three-year data were collected from ten centers across the US (Appendix 1). The current research is part of a post-approval study of Macroplastique® [Macroplastique® Real-time Observation of Safety and Effectiveness (ROSE) registry]. It is based on data lock-in August 2015 presented to FDA post-approval report P040050 on October 18, 2017.

Institutional review board (IRB) approval along with patient informed consent was obtained for this study. Inclusion criteria included: females ≥ 18 years of age, diagnosis of SUI due to ISD (confirmed by pelvic examination and urodynamics VLPP < 100 cmH_2_O), patient understanding of all the study material including the 5-year follow-up schedule, and patients who were psychologically stable and deemed suitable for the intervention by the investigator. Exclusion criteria included acute urinary tract infection/inflammation, pregnancy or intended pregnancy within 1 year, a sling placement within 12 weeks, a bulking agent within 12 weeks, bladder neck or urethral stricture, vaginal prolapse, untreated detrusor instability/overactivity, neurogenic bladder, or overflow incontinence.

The procedure was standardized for all centers per the protocol and performed by fellowship-trained FPMRS (urologist/urogynecologist); the MPQ was injected under local anesthesia or general sedation in either the OR or clinic. In brief, patients were placed in the lithotomy position, and MPQ was injected transurethrally into the submucosa 1.5 cm distal to the bladder neck under cystoscopic control using the Macroplastique Implantation System (MIS) at 6, 2, and 10 o’clock positions using the tunneling technique [7]. The patient was asked to void afterward. For patients unable to void within 6 h, a small straight catheter was utilized to empty the bladder.

Postoperative evaluations were scheduled at 3, 12, 24, and 36 months and then every year. The need for a second injection was a shared decision between the patient and the treating physician at least 3 months after the first injection. For patients receiving a second injection, the 3-year follow-up period started from then. Every follow-up visit included medical history, physical examination, and evaluation of patient satisfaction.

Subjective urinary continence outcome has been evaluated using the Stamey grade of urinary incontinence questionnaire (0 = continent, 1 = incontinence with vigorous activity, 2 = incontinence with minimal activity, and 3 = total incontinence) and Incontinence Quality of Life (IQOL) Questionnaire. IQOL is a 22-item, 5-point Likert-type self-reported quality of life measure specific to urinary conditions. It is divided into three subscales (Avoidance & Limiting Behavior, Psychosocial Impacts, Social Embarrassment), which were assessed at baseline, 12, 24, and 36 months post-injection. Satisfaction after injections was assessed by the Patient Global Impression of Satisfaction (PGI-S) at the 12-, 24-, and 36-month follow-up visits. Subjective success was defined in this study as an improvement to Stamey grade 0 or 1 at 36 months. Safety assessment was reported in terms of serious and non-serious adverse events (AEs).

The overall success rate and its 95% confidence interval were calculated as raw proportions. A linear mixed-effect model with patient-level random effect was used to examine longitudinal trends of the I-QoL and its subscales over the 3-year study period. Satisfactory scores and AEs were only summarized descriptively, with no statistical test used. All analyses were conducted using SAS software 9.4. *P*-values < 0.05 were considered statistically significant.

## Results

Of 70 patients followed and evaluated who completed 3-year follow-up from the last MPQ injection, 24/70 (34%) underwent two injections. The mean volume of MPQ used for the first injection was 4 ml (*n* = 70; range 1–10 ml), and 3.7 ml used for the second injection (range 2–10 ml).

**Fig. 1:**
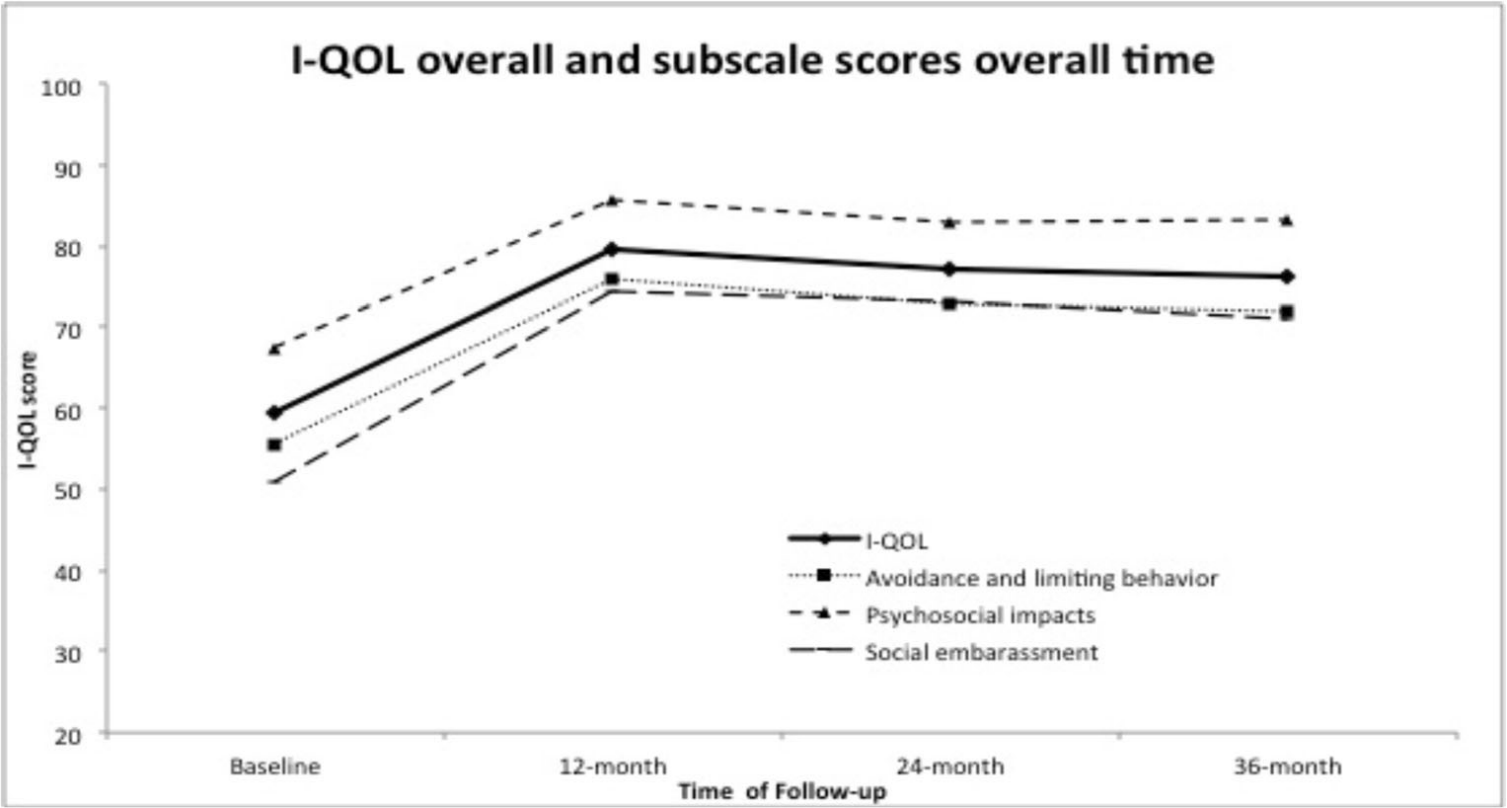
I-QOL scores and subscales significantly improved at 12, 24, and 36 months from baseline (*p* < 0.0001)

Baseline demographics are shown in Table 1. From these 70 patients who completed 3-year follow-up, 21/70 (30%) patients reported Stamey grade 0 and 28/70 (40%) had Stamey grade 1 (Table 2), while the overall satisfaction was 68%, and 27/70 (38.6%) patients reported they were very satisfied on PGI-S (Table 3). I-QoL scores and the subscales were significantly improved at 12, 24, and 36 months from baseline (*p* < 0.0001) and remained stable (Fig. 1 and Table 4).

**Table 1:** Baseline demographics of study participants

Characteristic	Mean ± SD (N)
Age (years)	63.3 ± 12 (70)
Weight (lbs)	179.5 ± 46.7 (70)
Height (inches)	72.5 ± 26(70)
BMI	28.5 ± 10.7(70)
Ethnicity percent (N)	
Hispanic	2.86(2)
White, not Hispanic	97.14(68)
Race	
White	94.29(66)
Asian	1.43(1)
American Indian or Alaskan Native	1.43(1)
Other	2.86(2)
Number of births	
0	10.29(7)
1	16.18(12)
2	35.29(25)
3	26.47(18)
4	7.35(5)
5	1.47(1)
6	1.47(1)
7	1.47 (1)
History of prior treatment in 55 patients	
Behavioral modification	27.27% (15)
Biofeedback	10.91% (6)
Mid-urethral sling	9.1% (5)
Surgical suspension (Burch)	12.73% (7)

**Table 2:** Stamey grade at baseline and annual follow-ups

Stamey grade	Baseline		12 months		24 months		36 months	
	N	%	N	%	N	%	N	%
0	0	0	12	17.65%	10	14.49%	21	30.00%
1	26	37.14%	40	58.82%	33	47.83%	28	40.00%
2	38	54.29%	12	17.65%	23	33.33%	19	27.14%
3	6	8.57%	4	5.88%	3	4.35%	2	2.86%
Total	70		68		69		70

**Table 3:** Patient global satisfaction (PGI-S) with MPQ treatment at 12, 24 and 36 months

	12 months n/N (%)	24 months n/N (%)	36 months n/N (%)
Very satisfied	32/68 (47.04%)	26/69 (37.7%)	27/70 (38.6%)
Somewhat satisfied	17/68 (25%)	23/69 (33.3%)	21/70 (30%)
Neither satisfied nor dissatisfied	7/68 (10.3%)	7/69 (10.14%)	7/70 (10%)
Somewhat dissatisfied	7/68 (10.3%)	7/69 (10.14%)	7/70 (10%)
Very dissatisfied	5/68 (7.3%)	6/69 (8.7%)	8/70 (11.4%)

Note: these 70 patients at 36 months are the same patients at 12 months

**Table 4:** QOL means and its subscales at baseline, 12, 24, and 36 months for the 70 patients who complete 3-year follow-up

	Baseline *n* = 70	12 months *n* = 68	24 months *n* = 69	36 months *n* = 70
I QoL	59.4 ± 19 [20,92.7]	79.6 ± 16.4 [35.4100]	77 ± 20 [30,100]	76.35 ± 20.5 [22.7100]
Subscale 1 (Avoidance)	55.6 ± 18.5 [20,95]	76 ± 17.7 [0.0, 100]	73 ± 21 [20,100]	72 ± 20.5 [25,100]
Subscale 2 (Social)	67.5 ± 22 [20,97.8]	85.7 ± 16 [33, 100]	83.1 ± 20 [22,100]	83 ± 20 [22,100]
Subscale 3 (Psychological)	60 ± 20.4 [20,92]	74.3 ± 16.3 [33, 100]	73.1 ± 22.2 [22,100]	71.2 ± 23.7 20,100]

Values are expressed as mean ± SD [minimum, maximum]

Transient dysuria (3.2%), hematuria (6%), pain at the injection site (1.6%), and urinary tract infection (2%) were the most common non-serious AEs that occurred within the first 3 months post-injection. No serious AEs were reported in these 3 years.

## Discussion

Evidence for the long-term efficacy/durability and safety of urethral bulking agents is limited. We report our results using Macroplastique® injection for the treatment of SUI in women who completed a 3-year follow-up from their last injection in a multicenter post-marketing study in the USA (ROSE Registry).

Polydimethylsiloxane (Macroplastique®, MPQ) is a UBA used in the treatment of SUI in women with ISD. It consists of soft, flexible, highly textured, irregularly shaped implants of heat-vulcanized polydimethylsiloxane (a solid silicone elastomer) suspended in hydro-carrier gel. The carrier gel is a pharmaceutical grade, water-soluble, low-molecular-weight polyvinylpyrrolidone (PVP or povidone) hydrogel, which is absorbed by the reticuloendothelial system and excreted unchanged in the urine [8].

In this study, we demonstrated stable overall satisfaction and improvement in urinary incontinence based on subjective assessments over 3 years. Side effects from MPQ injections were few and mild. Contrary to the belief that UBAs have temporary benefits, more recent data show long-term durable results [9, 10], which our data corroborate.

The outcome of incontinence treatments can be measured in different ways; a common one is the assessment of the need for a further incontinence procedure. We set stricter assessment criteria reflecting the clinical course after treatment. The composite outcome was determined by combining the patient-reported outcomes based on the questionnaires and some degree of improvement based on Stamey’s grade. None of these patients received a third injection or other anti-incontinence procedure. The use of a standardized questionnaire allows for longitudinal follow-up that has shown the sustainability of satisfaction over the years even if some subjects required repeat injection. Patients may have improved their urinary control and activities within their own Stamey grade, therefore accounting for a higher overall satisfaction compared to the composite success rate. It could be argued that objective parameters such as pad weight or urodynamic findings could be used to evaluate the success of UBAs, but our view is that use of validated questionnaires such as the IQOL and PGI-S are sufficient given that subjective perception of success is of greatest significance when it comes to treating conditions that affect the quality of life such as SUI.

Over the short term (12 months), MPQ efficacy is in the range of 35–80% [11]. Another study reported a 67% objective cure rate at 24 months in 75 women [8], and a smaller study (*n* = 21) reported a cure/improvement rate of 73% at 60 months [12]. At 20 months' median follow-up, MPQ improved subjective and objective outcome measures for SUI secondary to ISD as both a primary and secondary treatment option in women [13].

While the use of objective parameters might be necessary to verify the improvement of urine leakage when comparing interventions, the impact of these interventions on IQOL often does not correlate with objective measurements [14]. Addressing patient expectations and priorities is only achievable with robust information on subjective perceptions of therapeutic outcomes [15]. The IQOL improved significantly with UBAs compared to surgical procedures, despite the superior objective efficacy of surgery [16].

MPQ injections were also found to be safe, as no serious AEs occurred in this cohort and non-serious AEs were self-limiting. Treatment-related AEs were few, with mild and transient hematuria, dysuria, and UTI being the most common.

Two or three injections are likely to be required to achieve a satisfactory result [17]. Serati et al. [10] reported a learning curve showing significantly inferior efficacy for the initial 20 procedures, highlighting the need for more training in good ex-vivo models.

The cost-effectiveness of UBAs overall is not clear-cut but less expensive than tension-free vaginal tape, at least in the short term. Nevertheless, economic modeling suggests a higher cost for injection therapy in case multiple injections are needed [16] in the long term.

However, this may ultimately depend on the optimal selection of candidates for UBAs. Criteria for appropriate patient selection are still being debated but are an important consideration when planning the management of interventions for urinary incontinence in light of its impact on the quality of life. The minimal invasiveness, favorable safety profile, and new evidence of durable benefit and satisfaction at 3 years support the routine use of MPQ therapy [15]. Furthermore, UBA use appears not to jeopardize outcomes if future anti-incontinence surgery is needed [18]. On the other hand, UBAs can be used after failed MUS placement, with a lower objective cure rate but high patient satisfaction and no significant complications [5].

The strengths of this study are its prospective longitudinal design, use of a maximum of two injections, long-term monitoring, a large number of patients, and a large number of contributing centers reflecting real-world practice. A limitation of this study is the exclusion of patients who did not complete 3 years of follow-up and their outcomes. It was performed by different surgeons with different expert levels that could affect the outcomes. One could argue that including more objective measurements like pad weight may have allowed for a more quantitative outcome, but the durability and safety according to patient-perceived outcomes provide a stronger argument. Overall, the 3-year results of this study provide strong evidence of the sustained clinical benefit of MPQ and 5-year data will be reported in the future.

## Conclusion

At 3 years, the urethral bulking agent polydimethylsiloxane (Macroplastique®) was found to be safe and efficacious for the treatment of SUI secondary to ISD in women. The overall high satisfaction rate is sustained from baseline to 3 years post-injection. MPQ treatment for SUI/ISD is not temporary and should be considered in certain patients. Most complications were minor and transient without sequelae.

## Appendix 1

**Table 5:** Centers enrolled in the study

Site	Medical centers	City	State
1	University of Oklahoma	Oklahoma City	Oklahoma
2	University of California, Irvine	Orange	California
3	Urological Associates of Southern Arizona	Tucson	Arizona
4	University of California, San Diego	San Diego	California
5	University of Michigan Health Center	Ann Arbor	Michigan
6	Carolina Urology Partners	Huntersville,	North Carolina
7	Southern Urogynecology	West Columbia	South Carolina
8	Deaconess Clinic, Inc	Newburgh	Indiana
9	Western New York Urology	Cheektowaga	New York
10	The Florida Bladder Institute	Naples	Florida

## Abbreviations

MPQ: Polydimethylsiloxane (Macroplastique®)
SUI: Stress urinary incontinence
ISD: Intrinsic sphincter deficiency
UBA: Urethral bulking agent
QOL: Quality of life
I-QoL: Incontinence quality of life questionnaire
PGI-S: Patient global impression of satisfaction
AE: Adverse events
